# Repurposing of approved antivirals against dengue virus serotypes: an in silico and in vitro mechanistic study

**DOI:** 10.1007/s11030-023-10716-5

**Published:** 2023-08-26

**Authors:** S. H. Rashmi, K. Sai Disha, N. Sudheesh, Joseph Karunakaran, Alex Joseph, Anitha Jagadesh, P. P. Mudgal

**Affiliations:** 1 Manipal Institute of Virology, Manipal Academy of Higher Education, Manipal, India; 2 Department of Pharmaceutical Chemistry, Manipal College of Pharmaceutical Sciences, Manipal Academy of Higher Education, Manipal, India

**Keywords:** Dengue virus serotypes, Drug repurposing, RNA-dependent RNA polymerase, Molecular docking, Non-nucleoside inhibitors, Good health, well being

## Abstract

Dengue is an emerging, mosquito-borne viral disease of international public health concern. Dengue is endemic in more than 100 countries across the world. However, there are no clinically approved antivirals for its cure. Drug repurposing proves to be an efficient alternative to conventional drug discovery approaches in this regard, as approved drugs with an established safety profile are tested for new indications, which circumvents several time-consuming experiments. In the present study, eight approved RNA-dependent RNA polymerase inhibitors of Hepatitis C virus were virtually screened against the Dengue virus polymerase protein, and their antiviral activity was assessed in vitro. Schrödinger software was used for in silico screening, where the compounds were passed through several hierarchical filters. Among the eight compounds, dasabuvir was finally selected for in vitro cytotoxicity and antiviral screening. Cytotoxicity profiling of dasabuvir in Vero cells revealed changes in cellular morphology, cell aggregation, and detachment at 50 μM. Based on these results, four noncytotoxic concentrations of dasabuvir (0.1, 0.25, 0.5, and 1 µM) were selected for antiviral screening against DENV-2 under three experimental conditions: pre-infection, co-infection, and post-infection treatment, by plaque reduction assay. Viral plaques were reduced significantly (*p* < 0.05) in the co-infection and post-infection treatment regimens; however, no reduction was observed in the pretreatment group. This indicated a possible interference of dasabuvir with NS5 RdRp, as seen from in silico interaction studies, translating into a reduction in virus plaques. Such studies reiterate the usefulness of drug repurposing as a viable strategy in antiviral drug discovery.

**Graphical Abstract:**

In this drug repurposing study, dasabuvir, a known anti-hepatitis C drug, was selected through virtual screening and assessed for its anti-dengue activity.

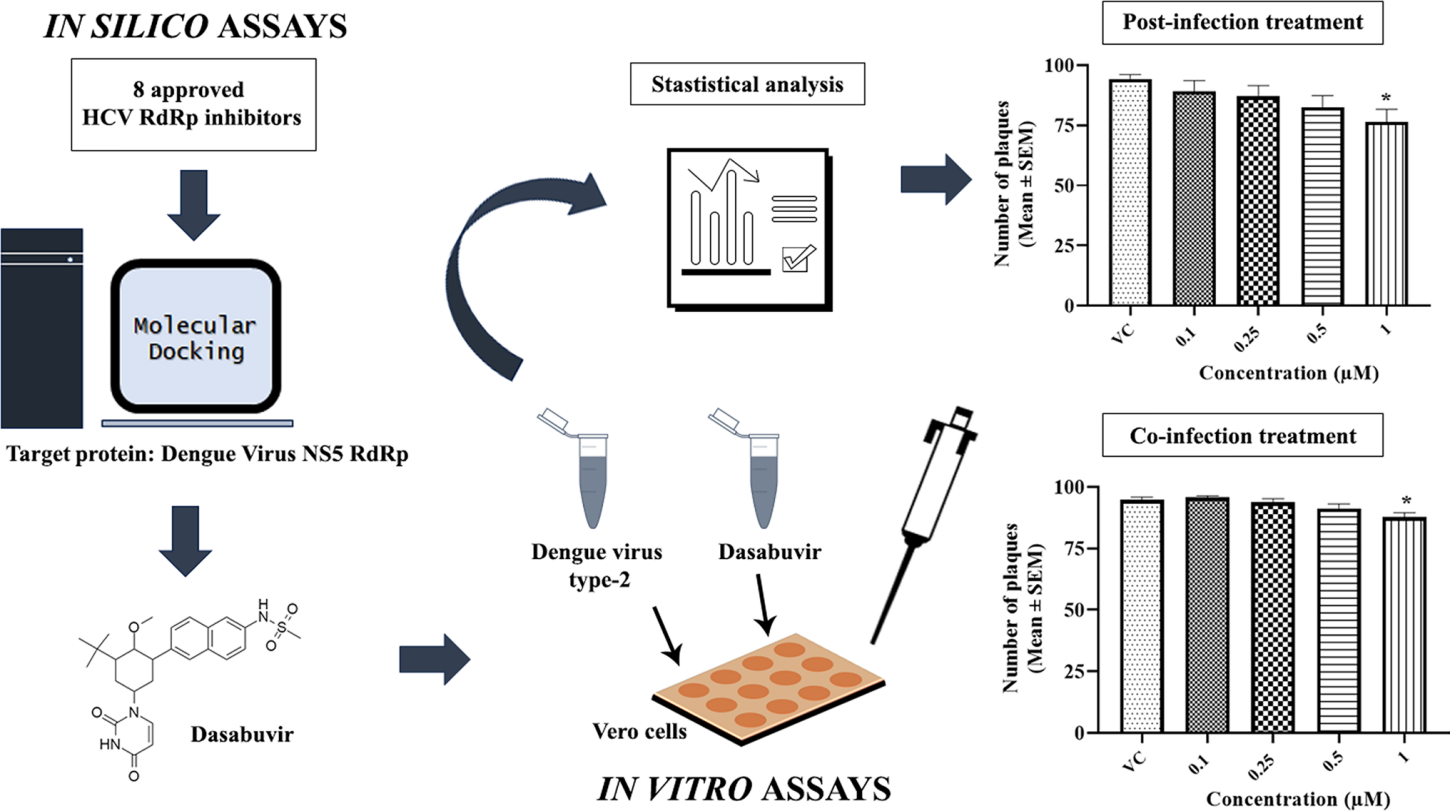

## Introduction

Dengue fever, a mosquito-borne viral disease, is caused by Dengue virus (DENV), a single-stranded, positive-sense RNA virus belonging to the family *Flaviviridae*. There are four serotypes of DENV, namely, DENV-1, 2, 3, and 4, which are genomically similar up to 65% [1]. Dengue viruses cause diseases ranging from asymptomatic or subclinical dengue fever to severe dengue marked by fluid leakage [2]. Dengue is endemic in the subtropical and tropical countries of Africa, Americas, Eastern Mediterranean, Southeast Asia, and Western Pacific [3]. Annually, with an approximate turnover of 390 million infections, which include 100 million apparent clinical cases, dengue has acquired a marked global presence, with a significant contribution by Southeast Asian and Western Pacific countries [4].

Despite concerted efforts, no clinically approved antiviral therapy against dengue is available to date, and the treatment is limited to symptomatic cure and supportive care in hospital settings [5]. Although, intravenous fluid supplementation is considered pivotal in dengue treatment, its effect on reducing fatality rate in severe cases is insignificant (< 1%)[6]. Thus, a need for safe and effective interventions to control this life-threatening disease is propelling research efforts towards discovery and development of promising molecules, before dengue turns into a catastrophe.

A spectrum of antiviral candidates have been designed and screened against dengue, but none has been successful in efficiently treating dengue infection [7, 8]. Recent advances in structural and molecular virology have contributed significantly to the identification of several viral enzymes as targets to design antiviral molecules [9]. In this context, conserved regions in the DENV genome encoding non-structural (NS) polymerase and protease enzymes have been identified as attractive targets in antiviral drug discovery. Conventional viral targets for the development of small molecule inhibitors include NS3 protease, NS3 helicase, NS4B, and NS5 proteins [10]. Of these, one of the extensively explored targets for antiviral development to date is the dengue non-structural protein NS5, due to its essential enzymatic and biological activities in the DENV replication process [11]. NS5 has methyltransferase (MTase) and RNA-dependent RNA polymerase (RdRp) activities encoded by the N-terminal end and the C-terminal end, respectively [12, 13]. NS5 protein binds to various host proteins during replication and inhibits the immune response of the host cell. NS5 protein is also responsible for inducing IL-8 secretion during severe dengue and has been found to bind to the STAT-2 molecule, thus stopping interferon production. Furthermore, the NS5 protein is the largest and most conserved with over 75% sequence homology across all four DENV serotypes [14, 15], conferring it as a potential target for the development of pan dengue antivirals.

New drug discovery is an expensive, tedious and time-consuming endeavor, thereby routing our attention to drug repurposing, which is an alternate, economically viable strategy to discover drugs for unmet medical conditions [16]. Repurposed drugs have a well-established safety and efficacy profile, that circumvents the need to carry out several studies of the preclinical and clinical phases. Reprofiling of clinically approved drugs against the multifunctional viral proteins of DENV could be beneficial in identifying novel inhibitors for the treatment of disease severity, thus extending a first-line approach for rare and neglected diseases. These diseases especially affect developing countries, where the conditions are difficult to address due to financial reasons, thereby implying the urgent need for an effective treatment. A majority of antiviral compounds function as inhibitors of viral polymerase and protease enzymes, as validated by the use of inhibitors against hepatitis B virus (HBV), hepatitis C virus (HCV), human herpes viruses, and human immunodeficiency virus (HIV) [17, 18]. Since flaviviruses are closely related to HCV [19], non-nucleoside analogues, especially those developed against HCV, have been tested against arthropod-borne flavivirus infections [20] and therefore could be promising candidates for repurposing in the treatment of dengue infections supported by experimental evidence. Based on the available knowledge, the current study was aimed at structure-based virtual screening of approved HCV RdRp inhibitors against the DENV target protein RdRp, followed by testing of in vitro anti-dengue activity in cell lines and analyzing the mechanistic correlation thereof.

## Materials and methods

### In silico screening

#### Selection of inhibitors using an in silico approach

The Maestro module of Schrödinger software (version 2018-3) was used to perform in silico molecular docking. Eight FDA-approved HCV non-nucleoside inhibitors were selected, namely, nesbuvir, sofosbuvir, filibuvir, mericitabine, dasabuvir, setrobuvir, valopicitabine, and tegobuvir, for in silico studies.

#### Protein preparation

The protein preparation wizard of the Maestro module of Schrödinger software was used to perform preliminary modifications to the protein. One of the prerequisites for performing in silico screening is the availability of a crystal structure of the protein [21]. The X-ray crystal structure of the RdRp enzyme of DENV-3, co-crystallized with an inhibitor, was imported from the Protein Data Bank (https://www.rcsb.org/structure/5JJR, PDB ID-5JJR). Preprocessing was performed using the Prime module. Co-factors were retained, and selected chains were modified to assign proper bond orders. All polar hydrogens were displayed, followed by optimization and minimization of the processed protein using default software settings [22]. The minimized protein was split into water and ligand. Using the default Glide setting, a grid was generated using the centroid of the co-crystallized ligand, with the default size of 20 Å. The scaling factor for the Van der Waals radii was set for the proteins at 1.0 with a partial charge lower than 0.25. This was done to specify the active sites within the minimized protein. To validate the docking protocol, the co-crystallized ligand was redocked to the protein. The minimized ligand and redocked ligand were superimposed to obtain the RMSD value.

#### Ligand preparation

Eight inhibitors of HCV RdRp were selected for docking. SMILES characters for the eight inhibitors were obtained from PubChem, and 2D structures were generated. These 2D structures were then converted to geometrically refined 3D structures using the LigPrep module in the software. The LigPrep module generates 3D structures with accurate chirality, original states of ionization, and tautomers or conformations by the Monte Carlo method using the OPLS3e force field [23].

#### Extra precision docking

The GLIDE module was used to dock the molecules. This module searches for suitable interactions between the ligand and active site of the protein through three-step hierarchical filters, namely, HTVS (high throughput virtual screening), SP (standard precision) and XP (extra precision). Ligand docking generates the conformations and orientations inside the binding pocket in the presence of Grid potentials. Extra precision (XP) docking was selected for the inhibitors, as the ligands were fewer in number. The protein was rigid, and the ligand was flexible [24–26].

#### Induced fit docking

Induced fit docking was performed for selected compounds. This docking protocol allows the protein and the ligand to be flexible. The first step was softened-potential Glide docking of the ligand against a rigid receptor. The minimized protein was selected, and the grid box was generated. The centroid of the ligand was selected for the position of the grid to be generated. Two grid boxes were automatically generated to distinguish between the ligand and the protein. Based on the ligand, the size and position of the grid box were generated automatically. The ligands were docked using Glide, and by default, twenty poses were created for each ligand.

The next step was protein structure prediction and minimization using the Prime module. The side chains were trimmed. Vander Waals scaling was set at default. After the structure was refined, twenty protein poses were generated for each ligand. The ligand was redocked using Glide against the low energy induced fit conformation from the previous step; however, only those structures that were within 30 kcal/mol were selected for this step. The final scores for these conformations were determined using GlideScore and Prime energy [27].

### In vitro screening

#### Compounds, cells, and virus

Of the two potential drug molecules, nesbuvir and dasabuvir, screened from in silico studies, nesbuvir was ruled out from the present study as it is no more a part of the HCV therapeutic regimen, owing to its hepatic toxicity [28]. Therefore, dasabuvir was taken further for the in vitro studies and was purchased from ChemScene LLC, New Jersey, USA, in powder form and stored at 2–8 °C.

Vero cells (ATCC® CCL-81™ strain) were cultured in Minimum Essential Media-Maintenance Media (MEM-MM, Gibco, Thermo Fisher Scientific, India), which was supplemented with 10% v/v Fetal Bovine Serum (FBS, Gibco, Thermo Fisher Scientific, India) and antibiotics. The stock of DENV-2 serotype, archived in the biorepository at Manipal Institute of Virology, was used for in vitro screening.

#### Cytotoxicity assay

Cytotoxicity was checked in Vero cells. Cells were seeded in a 96-well microtiter plate (cell culture grade, Nunc™, Thermo Fisher Scientific, India) and allowed to grow overnight. The initial stock of dasabuvir (Cat. No. CS-2028; M. Wt. 493.57) (10 mM) was prepared using 100% dimethyl sulfoxide (DMSO) and dissolved by sonication. From this, a working stock solution of 1 mM was prepared and used to make concentrations ranging from 1nM to 50 μM, which were further tested for cytotoxicity. The cells were observed under an inverted microscope for morphological changes such as degeneration, cytoplasmic vacuolation and granulation, and detachment of the cellular monolayer. After three days, the contents of the wells were discarded, and the cells were fixed with 70% v/v methanol and stained with 0.5% w/v crystal violet [29, 30].

#### Virus titration by plaque assay

Vero cells were seeded in a 12-well cell culture plate and allowed to grow overnight. Tenfold log dilutions of the virus were made in MEM-MM, added to respective wells and allowed to adsorb for 1 h at 37 °C with 5% CO_2_. The first overlay containing 1% agarose, 2X-Yeast extract – lactalbumin hydrolysate media (2x-Yelah) and sodium bicarbonate (7.5% v/v) was added after the adsorption period. The plate was incubated for 48 h. The second overlay [same composition as the first overlay + neutral red dye (0.3% v/v) for staining] was added after 48 h, and plaques were counted the next day.

#### Plaque reduction assay

A confluent monolayer of Vero cells in 12-well plates was used for the assays. DENV-2 was quantified using a plaque assay [31]. A plaque reduction assay (PRA) was performed to determine the antiviral activity of dasabuvir against dengue virus type-2 (DENV-2). Based on the literature, the virus titer used for PRA was 100 plaque forming units (PFU) [32]. PRA was performed using four noncytotoxic concentrations, 0.1, 0.25, 0.5, and 1 μM, of dasabuvir, tested in triplicate. The following treatment regimens were tried.

**Pre-infection treatment (pre-treatment with the drug)** Cells were treated with different concentrations of drug and incubated for one hour. Following this, virus was added to the cells and allowed to adsorb for one hour.

**Co-infection treatment (simultaneous addition of drug and virus)** Virus and drug were incubated for one hour. This mixture was added to the cells and incubated for one hour.

**Post-infection treatment (post-treatment with the drug)** Virus was added to the cells and allowed to adsorb, after which drug was added and incubated for one hour.

Following incubation with virus and drug in all three treatments, a plaque assay was performed as mentioned above. The number of plaques was counted for each drug concentration across the three treatment regimens.

#### Statistical analysis

The data from the plaque reduction assays are expressed as the mean ± SEM. One-way analysis of variance (ANOVA) followed by Dunnett’s multiple comparisons test was performed using GraphPad Prism software version 8.0.0 for Windows, www.graphpad.com, San Diego, California USA. A ‘*p*’ value of less than 0.05 was considered statistically significant.

## Results

### In silico studies

Eight inhibitors of HCV RdRp were virtually screened against the co-crystallized structure of DENV-3 RdRp. After superimposing the minimized ligand and the redocked ligand, an RMSD value of 0.25 Å was obtained, which validated the protocol.

#### Extra precision docking

LigPrep produced 19 conformers, which were docked against the energy minimized protein. The docking scores after XP docking ranged from − 5.9 to 0.7 kcal/mol (Table 1).

**Table 1:** Structures of eight selected ligands and their extra precision docking scores

Ligands	Structures	Docking scores (kcal/mol)	Types of Interactions and amino acid residues involved
Nesbuvir		− 5.960	3 hydrogen bonds (Arg737, Arg729, and one water molecule), 1 pi-cation bond (Arg729) and 1 pi-pi stacking (His711)
Sofosbuvir		− 5.283	3 hydrogen bonds (Arg457 and two water molecules) and 1 pi-cation bond (Arg729)
Filibuvir		− 4.490	4 hydrogen bonds (Gln339, Arg737, Thr794 and one water molecule) and 1 pi-cation bond (Arg729)
Dasabuvir		− 4.430	4 hydrogen bonds (Tyr758, Arg737, two water molecules), 1 pi-cation bond (Arg457) and 3 pi-pi stackings [Trp795 (3)]
Mericitabine		− 4.026	4 hydrogen bonds (Ser710, Arg737, two water molecules) and 1 pi-cation bond (Arg729)
Setrobuvir		− 3.846	4 hydrogen bonds (Arg737, Trp795 and two water molecules)
Valopicitabine		− 3.391	3 hydrogen bonds (Tyr758, Arg737, Ser318)
Tegobuvir		0.738	1 hydrogen bond (one water molecule), 1 pi-pi stacking (His711)

Of these eight inhibitors, nesbuvir and dasabuvir were selected for induced fit docking based on the nature of interactions and the number of hydrogen bonds, pi-pi stackings, and pi-cation bonds formed with the amino acid residues of the target protein, DENV-3 RdRp. These interactions increase the affinity of the ligand to the protein.

#### Induced fit docking

**Fig. 1:**
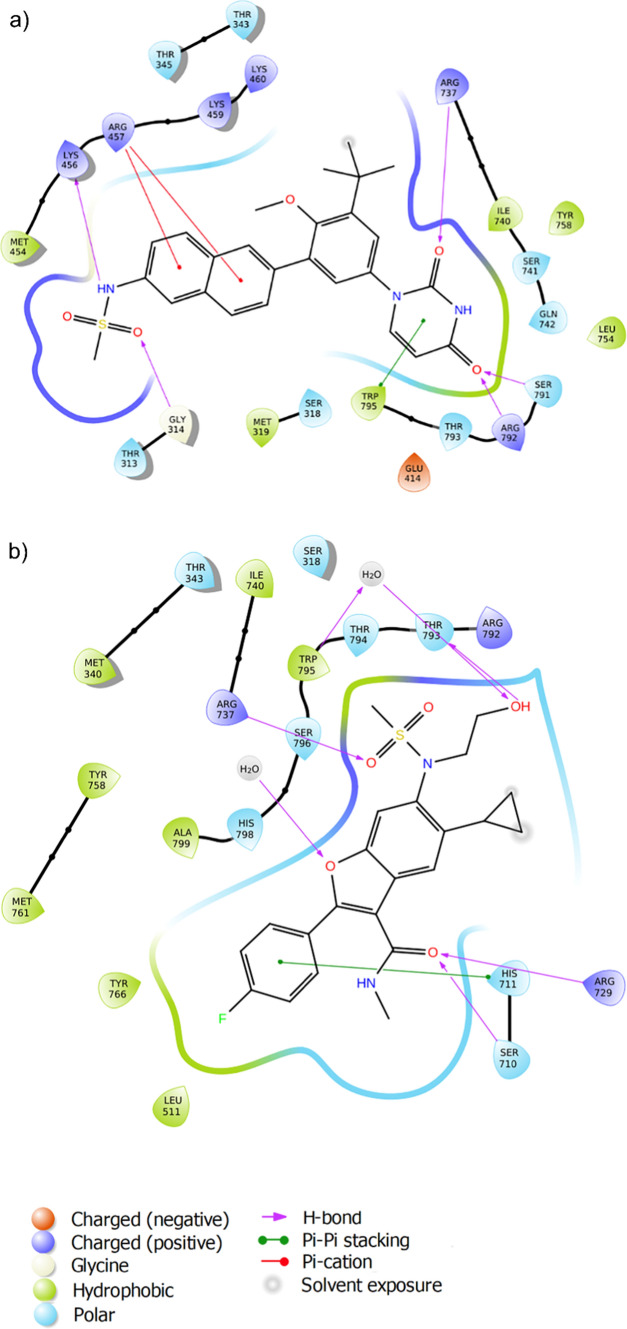
Post-docking (IFD) images of interactions between ligand and DENV RdRp protein (PDB ID: 5JJR) for two selected compounds. **a** Dasabuvir **b** Nesbuvir

**Fig. 2:**
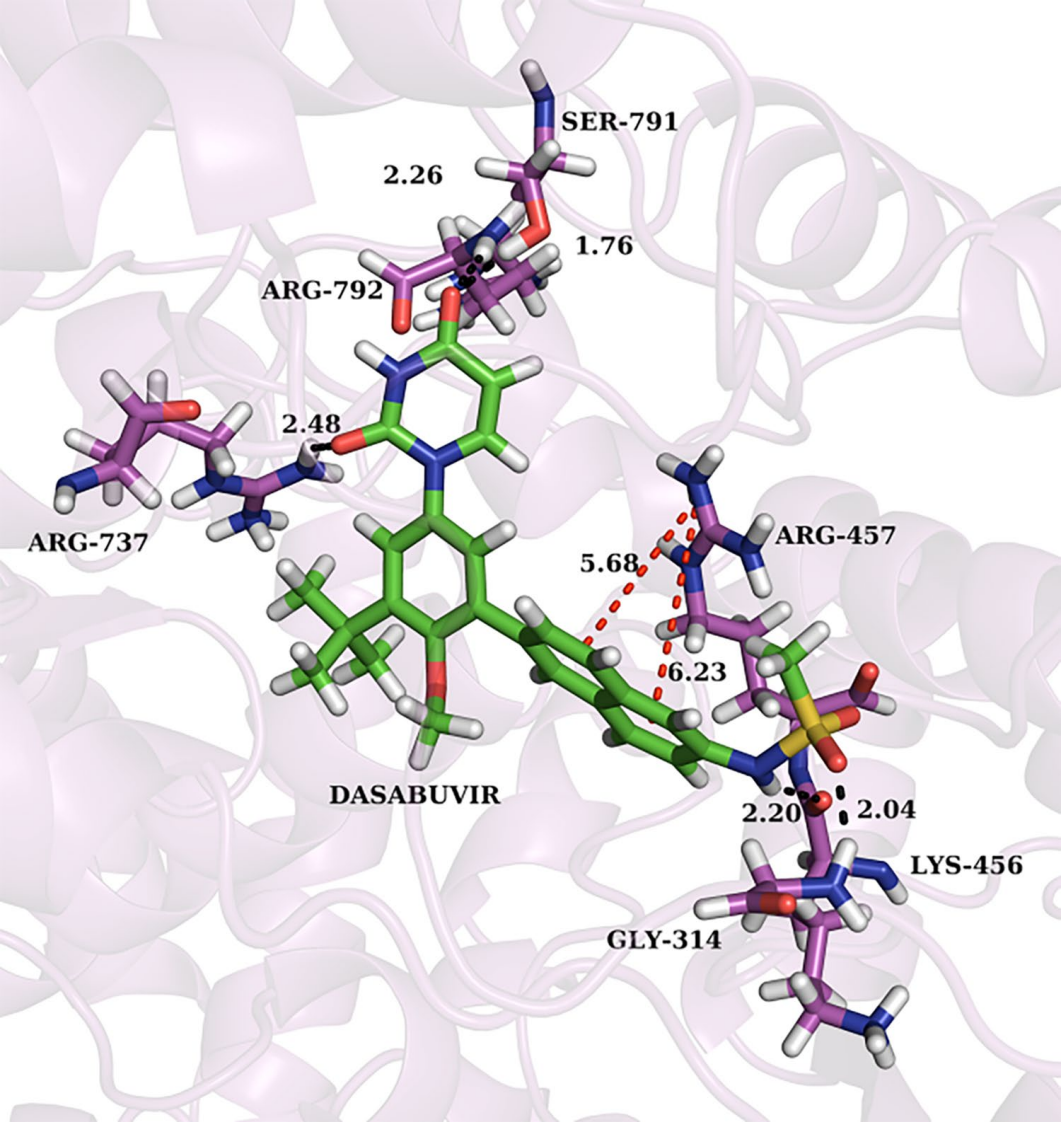
Complex of dasabuvir with DENV RdRp. PyMOL (http://www.pymol.org) was used to generate the 3D structure after performing induced fit docking. The image represents the best-docked pose for dasabuvir. The protein is depicted in the background as purple ribbons. Red-dotted lines represent pi-cation interactions. Black-dotted lines represent the hydrogen bonds. The bond lengths are measured in angstrom (Å) units

Dasabuvir interacted with the binding pocket of the protein in the following way. The 2,4 dioxo group of the pyrimidine ring in the dasabuvir structure interacted with Arg737, Ser791, and Arg792 through hydrogen bonds. Lys456 bound to the sulfonamide nitrogen through a hydrogen bond, while Gly314 interacted through a hydrogen bond with the oxygen atom of the sulfonamide group. The naphthalene ring of the ligand interacted with Arg457 through pi-cation bonds. There was only one site of solvent exposure towards the tertiary butyl group (Fig. 1a). The bonds and their bond lengths were further visualized with the help of PyMOL (Fig. 2).

Nesbuvir formed various bonds with the binding pocket of the protein. Arg729 and Ser710 bound to the oxygen atom of the amide side chain through two hydrogen bonds, while the hydroxyl group of the hydroxyethyl side chain interacted with Thr793 through another hydrogen bond. Trp795 was found to interact with the hydroxyl group of the hydroxyethyl side chain through a water molecule via hydrogen bonds. Arg737 interacted with the oxygen atom of a methyl-sulfonyl group through a hydrogen bond. The 4-fluorophenyl ring attached to the benzofuran ring interacted with His711 through a pi-pi stacking interaction. There were two sites of solvent exposure in the cyclopropyl ring (Fig. 1b).

### In vitro studies

#### Cytotoxicity assay

**Fig. 3:**
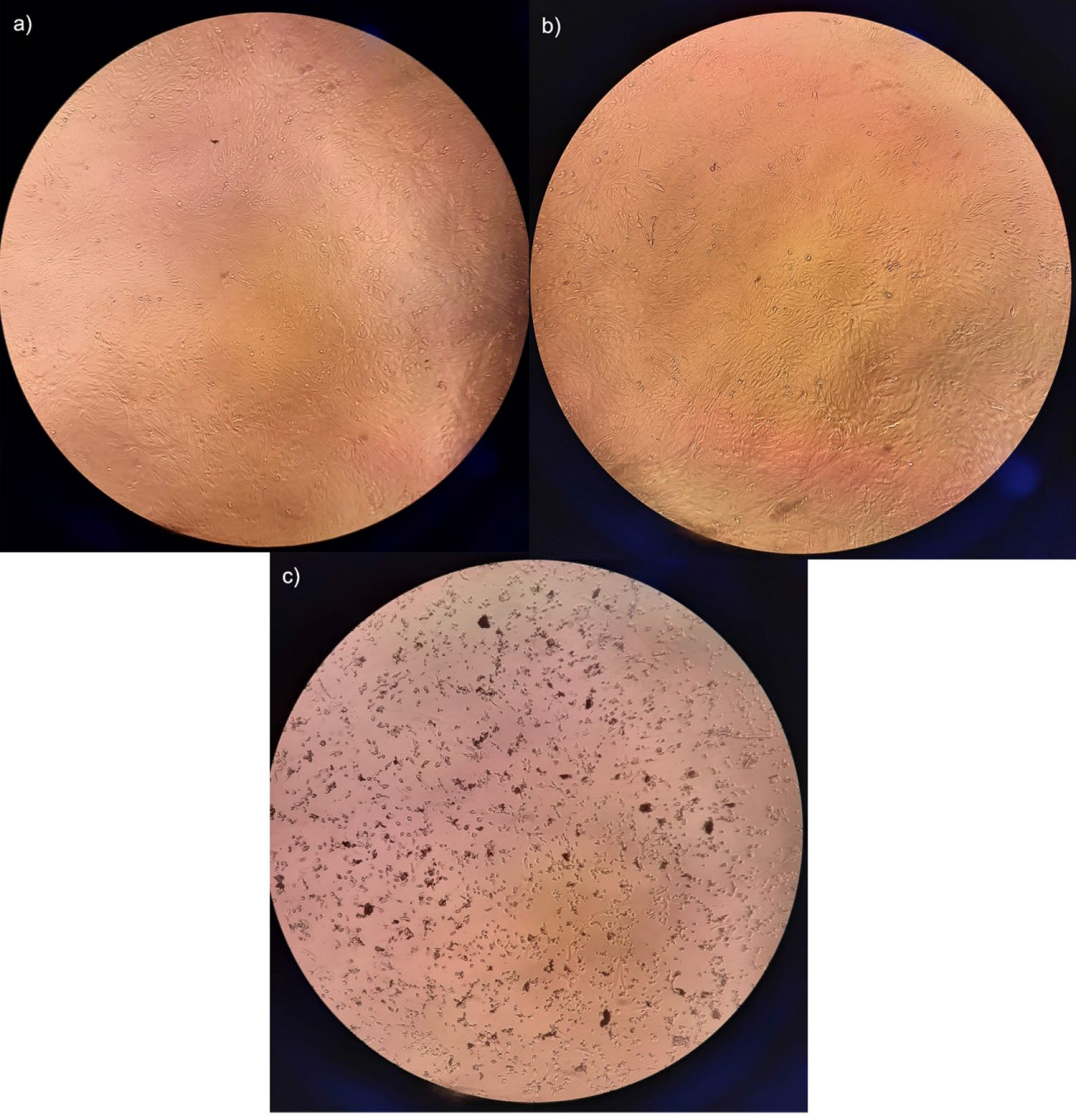
Cytotoxic effects induced by dasabuvir in Vero cells. **a** cell control; **b** non-cytotoxic concentration (10 μM); **c** cytotoxic concentration (50 μM). Morphological changes in the cells can be observed

The cytotoxicity of dasabuvir was determined based on morphological changes such as loss of cell integrity, shrinking of cells, aggregation, and detachment from the substrate, as observed microscopically (Fig. 3). The concentrations screened were 1 nM, 10 nM, 50 nM, 100 nM, 1 μM, 10 μM, 25 μM, 35 μM, and 50 μM. Cytopathic effects were evident at a concentration of 50 µM, below which there were no morphological changes. Based on the cytotoxicity assay results, a range of four noncytotoxic concentrations of dasabuvir were prepared and screened for antiviral activity against DENV-2.

#### Plaque reduction assay

Three independent experiments were carried out with four concentrations of dasabuvir, 0.1, 0.25, 0.5, and 1 μM. The plaques formed in the virus control and test wells are given below (Table 2).

**Table 2:** Plaque numbers for different concentrations of dasabuvir

Treatment groups	Number of plaques (Mean ± SEM)				
	Virus control	0.1 µM	0.25 µM	0.5 µM	1 µM
Pre-infection treatment	87.5 ± 5.3	86.5 ± 1.0	82.5 ± 1.7	91.5 ± 1.7	88 ± 1.4
Co-infection treatment	94.8 ± 1.0	95.8 ± 0.5	93.6 ± 1.5	91.3 ± 1.8	87.6 ± 1.8*
Post-infection treatment	94.3 ± 1.7	89.1 ± 4.4	87 ± 4.5	82.5 ± 4.8	76.5 ± 5.11*

The asterisk (*) indicates a significant difference (*p* < 0.05) in comparison with the virus control group.

**Fig. 4:**
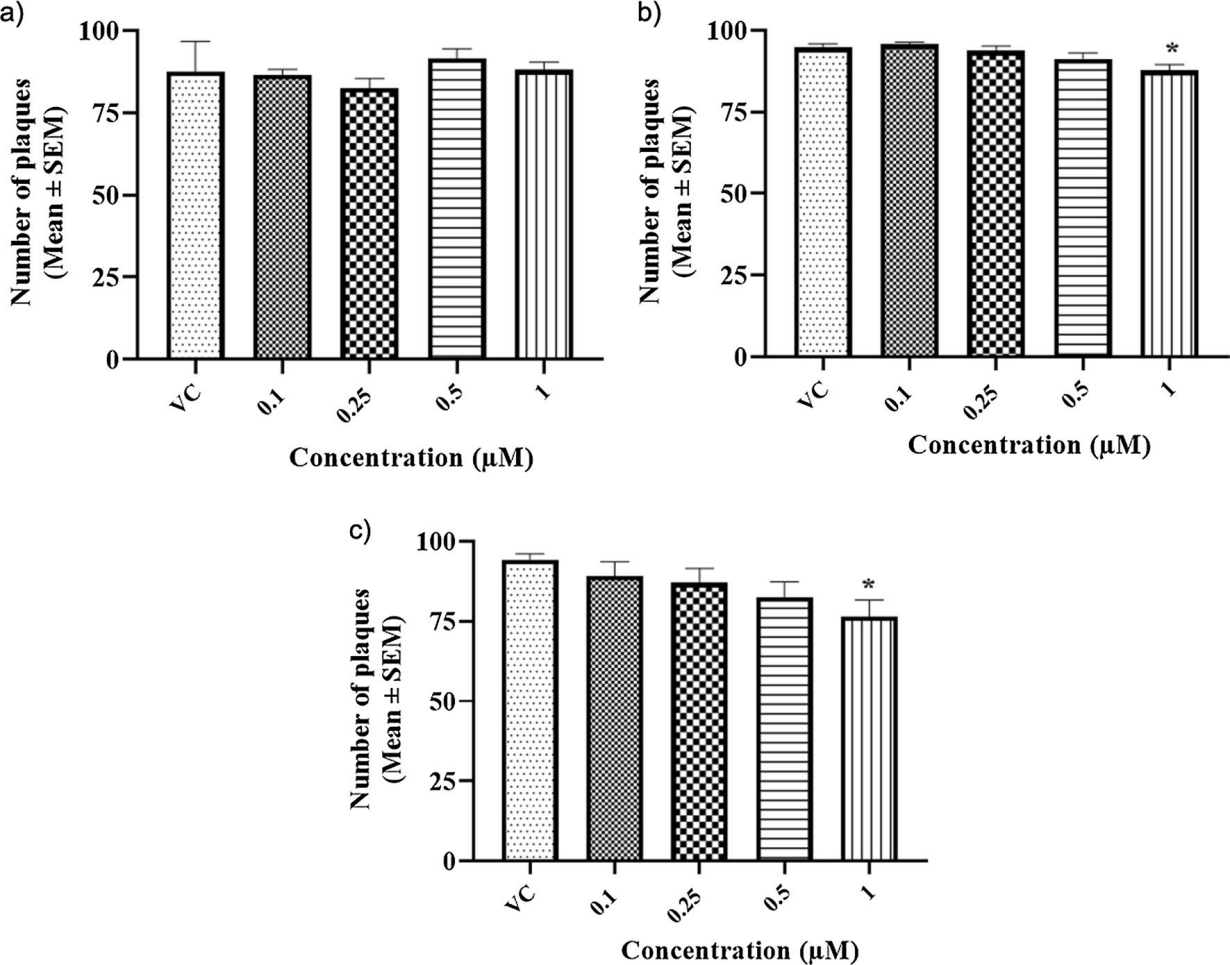
Results of plaque reduction assays for dasabuvir for three treatment regimens **a** pre-infection **b** co-infection **c** post-infection. The data were analyzed using GraphPad Prism version 8.0.0. Each bar represents the average plaque number (*n* = 3) for the three treatment regimens. The results are expressed as the mean ± SEM. The asterisk (*)indicates a significant difference (*p* < 0.05) in comparison with the virus control group (virus titer = 100 PFU)

There was no reduction in the number of plaques at any dasabuvir concentration compared to the virus control in the pre-infection treatment group. However, a significant reduction (*p* < 0.05) in plaque numbers was observed at 1 μM compared to the virus control both in the co-infection and post-infection treatment groups (Fig. 4). The percentage reduction translated into 7.6% and 18.9% in the co-infection and post-infection treatment groups, respectively. Below 1 μM, none of the concentrations of dasabuvir showed any significant reduction in plaque numbers compared to the virus control in either the co-infection or post-infection treatment groups.

## Discussion

The development of potent therapeutic agents is imperative to tackle the burden of dengue. Among the different approaches tried, virus target-based approaches to discover effective antivirals have yielded maximum hits. Dengue virus RdRp bears resemblance, both structurally and functionally, with that of HCV, except one difference that can be observed in the finger subdomain [19]. This structural similarity could be leveraged to screen a few existing HCV RdRp inhibitors against dengue virus as a drug repurposing approach in anti-dengue viral drug discovery.

Two major classes of RdRp inhibitors, originally developed against HCV, namely, nucleoside/nucleotide analogue inhibitors and non-nucleoside analogue inhibitors, have been investigated against DENV [33]. Earlier studies have revealed a first-in-class family of multitarget drug candidates able to inhibit DENV replication by acting on host kinases (Src/Fyn) and viral proteins (NS5-NS3 interaction). Among those candidates, four molecules, namely, nelfinavir, balapiravir, mycophenolic acid and ribavirin, are approved antivirals for other viral diseases [34]. Other studies on individual molecules revealed that sofosbuvir (GS-461203), an anti-HCV drug, exhibited excellent in vitro dengue virus inhibition with an EC_90_ of 0.4 μM and a binding affinity of -6.9 kcal/mol to the catalytic motif (Gly-Asp-Asp) of dengue viral polymerase [35]. The anti-influenza drug amantadine has also been found to be effective in limiting the growth of dengue viruses in cells [36], however the exact mechanism is undetermined. The nucleoside analogue of HCV, balapiravir, was tested in vitro and showed antiviral activity against DENV 1, 2, and 3. Furthermore, it was subjected to clinical trials, where it was found to be well tolerated. However, the treatment failed to reduce the viral load, NS1 antigenemia, and fever [7]. This laid the foundation for screening RdRp inhibitors of HCV against DENV NS5 RdRp through an in silico-driven approach, validated by in vitro methods in the present study.

Structure-based virtual screening, a computational technique used in drug discovery, is robust, useful and one of the most promising in silico techniques [37]. This technique is instrumental in rapidly searching libraries of small molecules based on their structural prerequisites, which will be helpful in binding to a drug target, typically a protein receptor or enzyme, and visualization of these interactions thereof. Repurposing drugs combined with virtual screening can speed up the drug discovery process. It is important to have crystal structures of proteins to successfully screen inhibitors [38–41]. With appropriate crystal structures and identification of various drug binding sites, novel antivirals can be designed, or existing antivirals can be repurposed for dengue virus. The reason for selecting a co-crystallized structure for molecular docking is the presence of a ligand bound to the protein, which defines the active site of the protein [42]. For this study, the co-crystallized structure of DENV-3 with an inhibitor (PDB-ID 5JJR) was used.

From computer-aided structure-based screening, dasabuvir showed good interactions with the RdRp of DENV, forming four hydrogen bonds, one pi-cation bond, and three pi-pi stacking bonds with the active site residues. Dasabuvir also formed the highest number of pi-pi stacking interactions among the other inhibitors. Pi-pi stacking bonds are non-covalent interactions, which are known to enhance the binding affinity of the ligand for the receptor protein [43]. Dasabuvir was discovered in a high throughput screening of aryl dihydrouracil derivatives that targeted the palm site of the HCV RdRp, encoded by the NS5 gene [44]. This molecule binds to the palm initiation pocket, thereby inducing conformational changes in the enzyme and inactivating it. The active site of DENV polymerase consists of Cys709, Ser710, His711, Arg729, Arg737, Tyr758, Thr793, Thr794, Thr795 and Ile797, which is also located in its palm domain [45]. This similarity in the location of the active sites in both enzymes was the rationale for selecting approved HCV RdRp inhibitors for molecular docking with dengue RdRp. These interactions indicated possible translation into anti-dengue viral activity, which led dasabuvir to be further tested in vitro for antiviral activity against DENV-2.

Before assessing the antiviral activity of dasabuvir, its cytotoxicity profile had to be established in Vero cells. In a previous study on Vero cells, dasabuvir was found to be moderately cytotoxic (CC_50_ value: 101.50 µM). In a replicon-based study, the 50% cytotoxic concentration (CC_50_) of dasabuvir was reported as 10.3 µM [46]. A study on cardiac Purkinje fibre cells found dasabuvir nontoxic up to 14 µg/mL, i.e., 28.3 µM [47, 48]. Based on the available literature, concentrations of dasabuvir ranging from 1 nM to 50 µM were tested for cytotoxicity. In the present study, dasabuvir per se did not show any toxicity in Vero cells up to 35 μM, above which toxicity was observed microscopically. At 50 µM, cytotoxicity was visible in the form of changes in cellular morphology, cell aggregation, and detachment from substrate by the end of the incubation period. The selective toxicity of dasabuvir could be a result of differential drug uptake by distinct cell types or its conversion into toxic metabolites by cellular enzymes [49].

Based on the cytotoxicity assay results and reported antiviral activity, four noncytotoxic concentrations of dasabuvir (0.1, 0.25, 0.5 and 1 µM) were selected for antiviral screening against DENV-2 under three experimental conditions: pre-infection, co-infection, and post-infection treatment. DENV-2 was used for in vitro antiviral studies considering its prevalence in India [50–53] as well as the severity of disease [54] caused by this serotype. A reduction (*p* < 0.05) in viral plaque numbers was observed in both co-infection (8%) and post-infection (19%) treatment regimens. The literature reports the effectiveness of dasabuvir against genotypes 1a and 1b of HCV at concentrations of 2.8 to 10.7 nM. In a replicon-based study [46] on HCV genotypes 2, 3, and 4, dasabuvir showed antiviral activity up to concentrations > 20 µM. In another study on FDA-approved antiviral drugs, efavirenz, tipranavir, and dasabuvir showed activity at micromolar concentrations against vector-borne flaviviruses [20].

The in vitro inhibition of DENV-2 by dasabuvir post-infection could be due to its interference with the functioning of multiple non-structural proteins, including the NS5 methyltransferase, NS5 RdRp, and NS2B-NS3 protease, thereby reducing viral replication [55]. This was correlated by in silico interaction studies of dasabuvir with the DENV NS5 RdRp enzyme. Post-docking visualization of the ligand‒protein interactions showed that dasabuvir bound to an active site residue, Arg737, and to other residues surrounding the active site, such as Arg792 and Ser791, through hydrogen bonds. The literature supports these hydrogen bond interactions responsible for effective binding of the molecules with the target protein, thereby inhibiting its activity.

Furthermore, dasabuvir exerts its antiviral activity by binding to allosteric sites present within the palm domain of HCV polymerase, which indicates an indirect mode of action. These regions mostly help by blocking the conformational changes that are required for the replication process [46, 56]. The palm I site of HCV RdRp is structurally equivalent to the N pocket, a known allosteric region in DENV RdRp [57, 58]. Residues Ser710, Arg729, and Arg737 are present within this pocket, and they play a significant role in the de novo replication process [56, 59]. The co-crystallized protein–ligand structure (PDB ID: 5JJR) used in the current study consists of an inhibitor (Compound 29), which is bound to significant amino acid residues lining the N pocket region of the DENV-3 RdRp. Since, the binding site for docking was defined by this N pocket region, the eight inhibitors in the current study were also seen interacting with the residues of the N pocket. Compound 29 primarily formed hydrogen bonds with the residues Arg729, Thr794, His800, and Gln802, which are part of the active site [58]. Although, structurally the selected molecules used in the current study are different from the compound 29, the interactions observed in both the studies are similar in terms of the hydrogen bonds formed with the active site residues, as observed in the XP docking results (Table 1). As mentioned earlier, Ser710, Arg729, and Arg737 line the N pocket of DENV-3 RdRp and are conserved across all members of the *Flavivirus* genus [58, 59]. Six of the eight inhibitors in the current study, except for tegobuvir and sofosbuvir, formed a hydrogen bond with Arg737. In case of dasabuvir, it retained its interactions with, Arg737, in addition to other residues as observed after flexible docking, which could explain the antiviral activity observed in the plaque reduction assay. Dasabuvir acts by blocking the nucleotide incorporation step in the replication cycle, thereby impairing the initiation process of the HCV polymerase. A similar mechanism can be suggested in this case as well, given the similarities between the viruses [28, 46].

In this work, only HCV inhibitors were screened against the RdRp of DENV. However, larger chemical databases, such as DrugBank or the Zinc database, could also be screened against the protein targets of DENV to obtain compounds with better interactions and docking scores. It would be interesting to explore the in vivo efficacy of dasabuvir against DENV in suitable animal models to validate its therapeutic prospects.

Certain studies have also suggested combining different classes of inhibitors with different modes of action against DENV. Synergistic antiviral activity has been observed in vitro and in vivo with promising results [46, 60, 61]. The main advantage of combining two different drugs is the possibility of administering the inhibitors at lower doses, thereby overcoming the issue of toxicity and other side effects [62]. Since dasabuvir is prescribed in combination with other inhibitors to treat HCV infection, a similar approach could be suggested in the case of dengue fever.

## Conclusion

Dasabuvir is a non-nucleoside inhibitor that is prescribed as part of a regimen for treating HCV infection. Owing to the similarities between the RdRp protein of HCV and DENV, a small library of RdRp inhibitors was virtually screened, and dasabuvir was selected. A plaque reduction assay was performed, and a significant reduction was seen at 1 µM in both co-infection and post-infection treatment with the compound. Although dasabuvir showed the potential to inhibit dengue viruses in the present study, using this molecule as an anti-dengue viral agent in isolation or in combination would require more experimental studies and data.
