# Correction to: Repurposing of approved antivirals against dengue virus serotypes: an in silico and in vitro mechanistic study

**DOI:** 10.1007/s11030-023-10748-x

**Published:** 2023-11-24

**Authors:** S. H. Rashmi, K. Sai Disha, N. Sudheesh, Kavitha Karunakaran, Alex Joseph, Anitha Jagadesh, P. P. Mudgal

**Affiliations:** 1 Manipal Institute of Virology, Manipal Academy of Higher Education, Manipal, India; 2 Department of Pharmaceutical Chemistry, Manipal College of Pharmaceutical Sciences, Manipal Academy of Higher Education, Manipal, India

**Correction to: Molecular Diversity (2023)** 10.1007/s11030-023-10716-5

In the original publication, one of the authors name was published incorrectly as “Joseph Karunakaran”. The correct name should read as “Kavitha Karunakaran”.

